# Diazotroph Genomes and Their Seasonal Dynamics in a Stratified Humic Bog Lake

**DOI:** 10.3389/fmicb.2020.01500

**Published:** 2020-07-01

**Authors:** Leyden Fernandez, Sari Peura, Alexander Eiler, Alexandra M. Linz, Katherine D. McMahon, Stefan Bertilsson

**Affiliations:** ^1^Department of Ecology and Genetics, Limnology and Science for Life Laboratory, Uppsala University, Uppsala, Sweden; ^2^Department of Forest Mycology and Plant Pathology, Science for Life Laboratory, Swedish University of Agricultural Sciences, Uppsala, Sweden; ^3^Centre for Biogeochemistry in the Anthropocene, Department of Biosciences, Section for Aquatic Biology and Toxicology, University of Oslo, Oslo, Norway; ^4^Great Lakes Bioenergy Research Center, Wisconsin Energy Institute, University of Wisconsin–Madison, Madison, WI, United States; ^5^Department of Bacteriology, University of Wisconsin–Madison, Madison, WI, United States; ^6^Department of Civil and Environmental Engineering, University of Wisconsin–Madison, Madison, WI, United States; ^7^Department of Aquatic Sciences and Assessment, Swedish University of Agricultural Sciences, Uppsala, Sweden

**Keywords:** diazotrophs, N-fixation, lake, hypolimnion, *nifH* gene

## Abstract

Aquatic N-fixation is generally associated with the growth and mass development of *Cyanobacteria* in nitrogen-deprived photic zones. However, sequenced genomes and environmental surveys suggest active aquatic N-fixation also by many non-cyanobacterial groups. Here, we revealed the seasonal variation and genomic diversity of potential N-fixers in a humic bog lake using metagenomic data and *nif* gene clusters analysis. Groups with diazotrophic operons were functionally divergent and included *Cholorobi*, *Geobacter*, *Desulfobacterales*, *Methylococcales*, and *Acidobacteria*. In addition to *nifH* (a gene that encodes the dinitrogenase reductase component of the molybdenum nitrogenase), we also identified sequences corresponding to vanadium and iron-only nitrogenase genes. Within the *Chlorobi* population, the nitrogenase (*nifH*) cluster was included in a well-structured retrotransposon. Furthermore, the presence of light-harvesting photosynthesis genes implies that anoxygenic photosynthesis may fuel nitrogen fixation under the prevailing low-irradiance conditions. The presence of *rnf* genes (related to the expression of H^+^/Na^+^-translocating ferredoxin: NAD+ oxidoreductase) in *Methylococcales* and *Desulfobacterales* suggests that other energy-generating processes may drive the costly N-fixation in the absence of photosynthesis. The highly reducing environment of the anoxic bottom layer of Trout Bog Lake may thus also provide a suitable niche for active N-fixers and primary producers. While future studies on the activity of these potential N-fixers are needed to clarify their role in freshwater nitrogen cycling, the metagenomic data presented here enabled an initial characterization of previously overlooked diazotrophs in freshwater biomes.

## Introduction

Recent studies in deep marine waters have revealed active N-fixation and a wide diversity of non-cyanobacterial N-fixers (Farnelid et al., 2013; Bombar et al., 2016; Gradoville et al., 2017). The metabolic process by which atmospheric dinitrogen gas (N_2_) is transformed to biologically more reactive forms is known as diazotrophy (Howarth et al., 1988). Despite the ecological and biogeochemical significance of this process, there is limited knowledge about the distribution of this trait in different freshwater microorganisms. In particular, very little is known about non-cyanobacterial diazotrophs in freshwater systems, while at the same time, many of these ecosystems feature steep redox gradients and anoxic zones that might provide unique habitats for previously unrecognized N-fixers.

Only select Archaea and Bacteria have the ability to transform and assimilate N_2_ gas, and the key enzyme to mediate this process is the nitrogenase (Canfield et al., 2010). Three nitrogenases co-exist in nature: molybdenum-iron (Mo-nitrogenase), vanadium-iron, and iron-only (Fe-only nitrogenase). The corresponding gene clusters involved are *nifHDK* (molybdenum-iron), *vnfHGDK* (vanadium-iron), and *anfHGDK* (Fe-only). Mo-nitrogenase is found in all diazotrophs, and its expression is regulated by Mo concentration. Vanadium-iron nitrogenase is present in some microorganisms and may be expressed when Mo is scarce. The other alternative nitrogenase, Fe-only, has dual enzymatic functions and may simultaneously reduce N_2_ and CO_2_ to CH_4_ and H_2_ in a single enzymatic step (Zheng et al., 2018).

As a result of metatranscriptomic oceanic surveys, significant expression of genes associated with N-fixation has been found in water below the photic zone. This implies a hitherto unrecognized non-cyanobacterial contribution to global marine N-fixation. The N-fixation activity of these non-cyanobacterial diazotrophs is typically associated with energy sources other than oxygenic photosynthesis (Farnelid et al., 2013). With the introduction of new high-throughput sequencing technologies and well-described marker genes such as *nifH* and *nifD*, the list of identified marine microorganisms potentially capable of N-fixation has grown in recent years. For instance, many proteobacterial diazotrophs (typically described as “heterotrophic N-fixers”) have been found to be abundant, widespread, and sometimes numerically dominant diazotrophs in the ocean (Bombar et al., 2016; Gradoville et al., 2017). In a N-fixation study carried out across the chemocline of the central Baltic Sea, two gammaproteobacterial-like *nifH* containing gene clusters, *EQF91* and *ALHOU*, were targeted by quantitative PCR (qPCR) and could be associated to active N-fixation despite ample availability of bio-assimilable N compounds (Farnelid et al., 2013). A more recent study conducted in the California Current System (Gradoville et al., 2017) identified representatives within the diazotrophic community from anaerobic members of *Deltaproteobacteria* and *Betaproteobacteria*, highlighting the variability in the composition and metabolic functioning of marine diazotrophs. Interestingly, non-cyanobacterial marine diazotrophs seem to fix N_2_ in waters where inorganic N concentrations were still sufficient to support growth (Bombar et al., 2016). This contradicts observations in earlier studies, in which *Cyanobacteria* appeared responsible for most planktonic N-fixation (Howarth et al., 1988) and may point to less constrained regulation of diazotrophy among the heterotrophic N-fixers.

Many questions remain regarding the broader role of N-fixers in freshwater ecosystems, the identity of important diazotrophs, and the metabolic traits enabling them to carry out the energy-demanding N-fixation. A pioneering study in Lake George, New York (Zani et al., 2000) attributed the active freshwater *nifH* phylotypes mainly to unicellular and filamentous *Cyanobacteria*, *Alphaproteobacteria*, and *Gammaproteobacteria*. A study carried out by Halm et al. (2009) in the Lake Cadagno (Switzerland), reported for the first time that green sulfur bacteria (GSB), namely, *Chlorobi*, could be involved in freshwater N-fixation. In this study, they also targeted *nifH* genes expressed as mRNA, showing that N-fixers were more active at the chemocline. By *in situ* hybridization-secondary ion mass spectroscopy, they further verified active diazotrophy in *Chlorobi*. Focusing on deep lake sediments rather than the overlying waters, Parro et al. (2017) also recorded the presence of nitrogen fixation gene products using immunodetection and hypothesized that these were associated with non-cyanobacterial diazotrophs such as Azospira and Arcobacter seen in 16S rRNA inventories. Further, a phylogenetically diverse group of non-cyanobacterial diazotrophs was recently suggested to dominate N-fixation in permafrost thaw ponds, and were possibly engaged in syntrophic relationship with methanogenic *Archaea* residing in these ponds (Fernandez et al., 2019). However, many freshwater diazotrophs, particularly those inhabiting the darker waters of lakes, have remained unexplored.

Here, we studied the temporal dynamics of potential diazotrophs by analyzing metagenomic sequence data and environmental parameters from epilimnetic and hypolimnetic waters of Trout Bog Lake (Wisconsin, United States), representing a lake type typical for boreal regions. This direct sequencing approach avoids the pitfalls and limitations introduced by primer coverage and other inevitable biases related to PCR-based assays. The samples were collected from early spring to autumn across three consecutive years, providing the first broader account of the temporal dynamics of diazotrophs in humic lakes. We also describe the main genomic features of the most abundant diazotrophs in the anoxic bottom waters (hypolimnion) of Trout Bog Lake by analyzing metagenome assembled genomes (MAGs). The thermal stratification in the small and sheltered Trout Bog Lake is very stable during the warm period of the year. Furthermore, the water has high allochthonous dissolved organic carbon concentration, and thus, light penetration is limited to a shallow upper layer (Baines et al., 2000). The deep waters in stably stratified freshwater lakes are frequently anoxic for extended periods of time (depending on the oxygen demand associated with microbial respiration) until brief mixing events may inject oxygen during the spring and autumn. Such anoxia may favor N-fixing activity, as oxygen is known to be a potent inactivator of nitrogenases. Despite the fact that high N-fixation capacity is often associated with eutrophication, Linz et al. (2018) calculated significantly higher levels of nitrogenase marker genes in the hypolimnion of Trout Bog Lake compared to the eutrophic and highly productive Lake Mendota (also located in Wisconsin, United States). This finding corroborates that seasonally anoxic hypolimnetic waters of humic lakes (such as Trout Bog Lake) may well be an overlooked habitat for N-fixers. Thus, based on genomic data, we aimed to also analyze alternative energy production pathways which may potentially fuel N-fixation in such light limited environments.

## Materials and Methods

### Trout Bog Lake Dataset

We included metagenome data from 42 epilimnetic and 45 hypolimnetic samples corresponding to the project Trout Bog Hypolimnion and Epilimnion Combined Assembly (Project JGI-ID: 416375), downloaded from the Department of Energy Joint Genome Institute (JGI) Genome Portal (Grigoriev et al., 2011). The data consisted of paired-end Illumina reads (average length approximately 200 bp after merging forward and reverse reads) (Bendall et al., 2016). The samples were collected from spring to autumn (or late summer depending on the year). The time series spanned a period from 2007 to 2009. Temperature and dissolved oxygen were measured throughout the sampling campaigns. In summary, the water temperature in the hypolimnion was approximately 5°C during the collection period, and suboxic conditions prevailed (dissolved oxygen concentration below 0.5 mg l^–1^ in most of the samples). More detailed information on the environmental data from Trout Bog’s epilimnion and hypolimnion can be found in previous work by Linz et al. (2017) and Table 1.

**TABLE 1:** Environmental data from 2008.

Date	TN (ppb)^a^	TP (ppb)^b^	TDN (ppb)^c^	TDP (ppb)^d^	Sample
29MAY08	575	19	479	12	Epilimnion
13JUN08	551	23	447	11	Epilimnion
24JUN08	629	31	417	15	Epilimnion
08JUL08	678	134	548	109	Epilimnion
22JUL08	1187	61	1019	38	Epilimnion
05AUG08	758	38	509	17	Epilimnion
19AUG08	865	46	585	22	Epilimnion
19SEP08	679	40	–	–	Epilimnion
29MAY08	1049	43	456	15	Hypolimnion
13JUN08	1152	49	987	35	Hypolimnion
24JUN08	1140	53	853	35	Hypolimnion
08JUL08	1161	63	999	42	Hypolimnion
22JUL08	684	36	527	15	Hypolimnion
05AUG08	1152	57	895	34	Hypolimnion
19AUG08	1117	65	1024.5	45	Hypolimnion
19SEP08	1513	60			Hypolimnion

*^a^Total nitrogen (TN) in parts per billion. ^b^Total phosphorus (TP) in parts per billion. ^c^Total dissolved nitrogen (TDN) in parts per billion. ^d^Total dissolved phosphorus (TDP) in parts per billion.*

### Enumeration of *nifH* Using Genomes in JGI-IMG

To measure *nifH* abundances, we used merged but unassembled reads. We compared all reads to genomes in JGI-IMG, including previously published MAGs from Trout Bog Lake (Bendall et al., 2016). These MAGs were assembled by Bendall et al. (2016) using metagenomes from a seasonal sampling over three consecutive years at Trout Bog Lake. Their assembly method was a “combined assembly” with samples pooled by layer (epilimnion or hypolimnion) before assembly (Bendall et al., 2016; Linz et al., 2018). Binning contigs into MAGs was based on differential coverage and kmer frequencies using MetaBAT (Kang et al., 2015). All these genomes were previously annotated using IMG tools (Chen et al., 2017) and we are using the naming of the MAGs as in the JGI database (Composite genome 1 to Composite genome 4645).

Our estimates of *nifH* gene frequencies in the communities were based on searches against reference genomes, MAGs (Bendall et al., 2016) and single amplified genomes (SAGs) from JGI. First, we used MAGs derived from Trout Bog Lake as the subject database. We then amended this with other reference genomes included in the JGI-IMG database (version March 2017) to identify *nifH* genes that were not represented in the previously assembled Trout Bog MAGs. These had been assembled by Bendall et al. (2016) using the same metagenomic library from Trout Bog Lake that was used for *nifH* screening in the current work. Nitrogenase gene annotations were compared to Pfam (Finn et al., 2014), KEGG Orthology (KO) (Kanehisa et al., 2016), and COG-based annotations downloaded for JGI-IMG. If any of these annotations pointed to a different function, all reads recruiting to that gene were excluded from further analysis. The relative *nifH* frequency was estimated as the total number of *nifH* hits compared to the total number of sequences used as input (metagenomic library size). This procedure was applied to calculate the proportions of nitrogenase genes in both epilimnetic and hypolimnetic samples. If the identified *nifH* sequence did not match any of the Trout Bog MAGs (Bendall et al., 2016), our implementation of the *nifH* enumeration method repeated the recruitment using the entire JGI-IMG collection of isolate genomes, MAGs, and SAGs as references. To retrieve taxonomic information about the reads, only high-confidence predictions were considered, using e-value = 10^–20^ as a cut-off and a percentage of identity = 95%. When several BLAST hits were exceeding these cut-off values, the taxonomic assignment of the hit with the highest percentage of identity and the lowest e-value was used. Thus, only one taxonomic classification was assigned per metagenomic read to avoid multiple counts. MAGs assembled using metagenomic data from Trout Bog Lake, as well as isolate genomes and SAGs from the entire IMG collection, where at least one copy of a putative *nifH* gene was found, are listed in Supplementary Table S1. Less than 1% of the reads assigned to a nitrogenase gene could not be classified in the taxonomic analysis.

### The Collection of Nitrogenase Positive Metagenome Assembled Genomes

To identify MAGs carrying nitrogenase genes (hereafter called nitrogenase positive MAGs), we used nitrogenase gene annotations as described above for *nifH* gene frequency analysis. In brief, gene sequences within the bin had to be classified as a *nifH* gene by all three annotation systems (KO, PFAM, and COG) (% identity > 95%) for the MAG to be considered a nitrogenase positive genome.

For further analysis, we selected representative genome bins where *nifH* abundances exceeded 50 *nifH* reads per phylotype, summed across the Trout Bog Lake hypolimnion time series from 2007 to 2009 (Supplementary Table S2). Our estimate of the abundances of *nifH* genes in the communities was based on BLASTp searches (cut off: 95% of identity) against MAGs (Bendall et al., 2016) derived from Trout Bog Lake as the subject database. The relative *nifH* frequency was estimated as the total number of *nifH* assigned hits compared to the total number of sequences used as input. Two MAGs with high *nifH* abundances in the time series from 2007 to 2009 were assembled from epilimnion data [i.e., Composite genome 211 (scaffold: TE211DRAFT TBepi metabat 211 1000175.54) and Composite genome 2493 (scaffold: TE2493DRAFT TBepi metabat 2493 1001483.68)]. However, the rest of the MAGs analyzed here (15 MAGs) were assembled using DNA data collected from Trout Bog hypolimnion samples (see Supplementary Table S2 and the scaffolds used for nitrogenase gene clusters reconstruction listed below). In addition, we checked for genome completeness to select high quality MAGs for whole genome analysis to determine functional capabilities of the contributing organisms (Table 2 and Supplementary Table S2). KEGG annotation system was used for the whole genome analysis (Supplementary Files in https://github.com/microbioinformatic/Trout-Bog-diazotrophs).

**TABLE 2:** JGI-IMG information about the MAGs used in the whole genome analysis.

Genome	Affiliation (IMG)*	Genome completeness (%)	IMG* genome ID
Composite genome 254	Bacteria; Acidobacteria	80	2582580662
Composite genome 4645	Bacteria; Proteobacteria	88	2582580689
Composite genome 433	Bacteria; Proteobacteria	77	2582580684
Composite genome 2922.v2	Bacteria; Proteobacteria	93	2593339183
Composite genome 111	Bacteria; Chlorobi	92	2582580651
Composite genome 211	Bacteria; Chlorobi	94	2582580622
Composite genome 3520v2	Bacteria; Chlorobi	89	2593339184
Composite genome 2493	Bacteria; Chlorobi	89	2582580625

*JGI’s Integrated Microbial Genomes (IMG) database.

In addition to the phylogenetic description provided by JGI-IMG and the information included in a previous publication (Linz et al., 2018), PhyloPhlAn (Segata et al., 2013) was used to improve phylogenetic and taxonomic classification for some poorly characterized putative diazotrophic MAGs downloaded from JGI-IMG (Supplementary Table S2). Interactive tree of life (iTOL) was used for phylogenetic tree visualization (Letunic and Bork, 2016). To build the tree, we also included other relevant genomes downloaded from JGI-IMG as references.

### Nitrogenase Gene Clusters Reconstruction

Genomic features analysis and neighborhood analysis of the *nifH* genes for the MAGs were done and visualized using Easyfig (Sullivan et al., 2011). To create GenBank annotation files for the CoDing Sequences (CDSs) needed for input to Easyfig, we used EMBOSS 6.6.0 release (seqret). With this tool, we converted the generic feature format (GFF) files downloaded from the JGI Genome Portal to GenBank format.

The scaffolds included in the GFF files and used to analyze different *nifH* operon structures were:

*Chlorobi*: (I) Composite genome 111 (scaffold: TH111DRAFT TBhypo metabat 111 10000140.1), (II) Composite genome 211 (scaffold: TE211DRAFT TBepi metabat 211 1000175.54), (III) Composite genome 2493 (scaffold: TE2493DRAFT TBepi metabat 2493 1001483.68), (IV) Composite genome 3520v2 (scaffold: TH03520DRAFT TH03520 TBL comb47 HYPODRAFT 10004746.48).

*Acidobacteria*: Composite genome 254 (scaffold: TH254DRAFT TBhypo metabat 254 10001420.155). *Sulfurimonas*: Composite genome 1998 (scaffold: TH1998DRAFT TBhypo metabat 1998 10000844.40). *Verrucomicrobia*: (I) Composite genome 2747 (scaffold: TH2747DRAFT TBhypo metabat 2747 10008023.254) and (II) Composite genome 2270v2 (scaffold: TH02270DRAFT TH02270 TBL comb47 HYPODRAFT 10000592.10).

*Methylococcales*: (I) Composite genome 3552 [scaffolds: (A) TH3552DRAFT TBhypo metabat 3552 10001230.12, (B) TH3552DRAFT TBhypo metabat 3552 10006655.34] and Composite genome 2062v2 [scaffolds: (A) TH02062DRAFT TH02062 TBL comb47 HYPODRAFT 10005551.84, (B) TH02062DRAFT TH02062 TBL comb47 HYPODRAFT 10000828.15].

*Deltaproteobacteria*: (I) Composite genome 4645 (scaffold: TH4645DRAFT TBhypo metabat 4645 10001890.329), (II) Composite genome 2922v2 (scaffold: TH02922DRAFT TH02922 TBL comb47 HYPODRAFT 10000417.24), (III) Composite genome 433 (scaffold: TH433DRAFT TBhypo metabat 433 10016042.145).

*Betaproteobacteria*: (I) Composite genome 2160 (scaffold: TH2160DRAFT TBhypo metabat 2160 10003883.171), (II) Composite genome 2159 (scaffold: TH2159DRAFT TBhypo metabat 2159 10000110.67).

### Statistical Analyses

All the statistical analyses were performed in R version 3.3.2 (2016-10-31) (R Core Team, 2016). For data visualization, we used the package ggplot2 (Wickham, 2009). The R scripts used to generate the main figures and statistical analysis are available at https://github.com/microbioinformatic/Trout-Bog.

## Results and Discussion

As information about freshwater diazotrophs is scarce, particularly in non-photic zones, we analyzed *nifH* seasonal variation and classified *nifH* positive MAGs to Trout Bog Lake time series samples. With these analyses, we identified the potential N-fixing taxa common in the hypolimnion. Additionally, we examined the *nif* operon of the main taxonomic groups to catalog genes with a primary role in N-fixation.

### Identity of Diazotrophs

We identified eight main phylogenetic groups from Trout Bog hypolimnetic water samples accounting for most of the observed *nifH* gene phylotypes (see section “Materials and Methods”). The main phylotypes corresponded to (1) *Sulfurimonas* within the *Epsilonproteobacteria*, (2) *Geobacter* and (3) *Desulfobacterales* within *Deltaproteobacteria*, (4) *Methylococcales* within *Gammaproteobacteria*, as well as (5) *Acidobacteria*, (6) *Betaproteobacteria*, (7) *Verrucomicrobia*, and (8) *Chlorobi* (Supplementary Figure S1).

### Seasonal Shifts in *nifH* Gene Abundances and the Relationship to Environmental Factors

**FIGURE 1:**
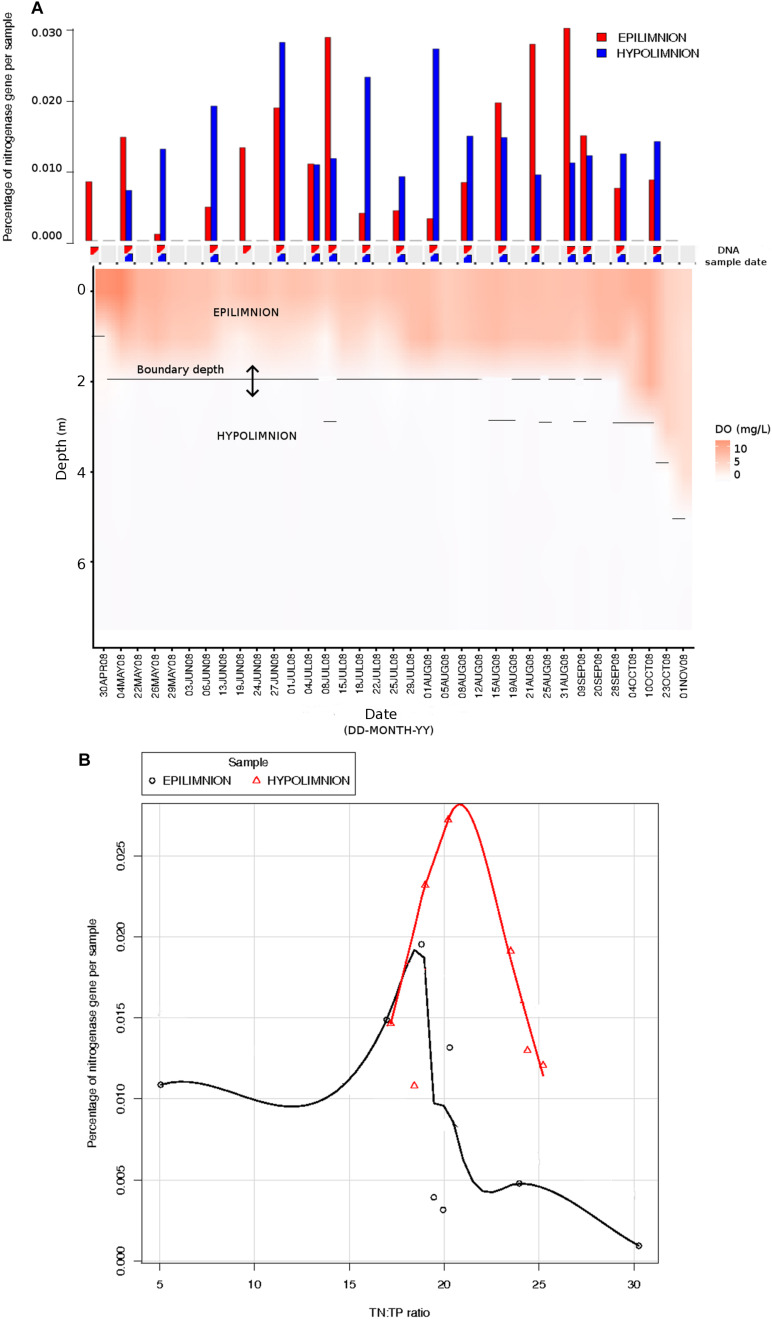
Comparison between nitrogenase gene abundances calculated from epilimnion and hypolimnion and environmental data during 2008 sampling campaign. **(A)** Nitrogenase gene percentages calculated using IMG annotations (bar plot: in red epilimnion and blue hypolimnion). The approximate depth and date when the DNA samples were collected are depicted with triangles (red = epilimnion and blue = hypolimnion). Dissolved oxygen (mg/L) is showed as a continuous heat map. **(B)** The relationship between TN:TP ratio (measured in part per billion) and the abundance of nitrogenase genes calculated from epilimnion and hypolimnion using IMG annotations. The polynomial function of non-linear regression is shown in red for hypolimnion and in black for epilimnion.

The relationship between *nifH* abundances and environmental conditions were analyzed for year 2008, as this was the only year for which the environmental data exist. Potential diazotrophs in the epilimnion and hypolimnion showed similar maximum abundances (∼0.030% nitrogenase genes per sample) during 2008 (Figure 1). The nitrogenase gene abundances were not related to dissolved oxygen and peaks in nitrogenase gene frequencies occurred independently of oxic/anoxic conditions (Figure 1A). However, the relative abundance of potential N-fixers in individual epilimnetic samples usually reached values exceeding 0.025% (nitrogenase relative total reads) on occasions when average DO was lower than the epilimnetic average (heat map in Figure 1A). The position of the chemocline occasionally compromised the collected epilimnetic samples, causing an apparent reduction in average DO levels. For instance, the upper 2–3 m layer of the anoxic bottom water was included in the supposedly epilimnetic DNA sample on 15 July 2008, 25 August 2008, and 9 September 2008. The seasonal shifts observed within the potential N-fixers in epilimnion and hypolimnion abundances were not significantly linearly correlated to any other environmental parameters measured, including TN, TDN, TP, and TDP (Figure 1 and Table 1). Interestingly, TN:TP ratio and nitrogenase gene abundances followed a polynomial function, with a turning point at a TN:TP of around 20 for both the epilimnion and hypolimnion. This may suggest a response of N-fixers to the TN:TP ratio in this humic lake (Figure 1B), independent of sampling depth and oxygen availability.

**FIGURE 2:**
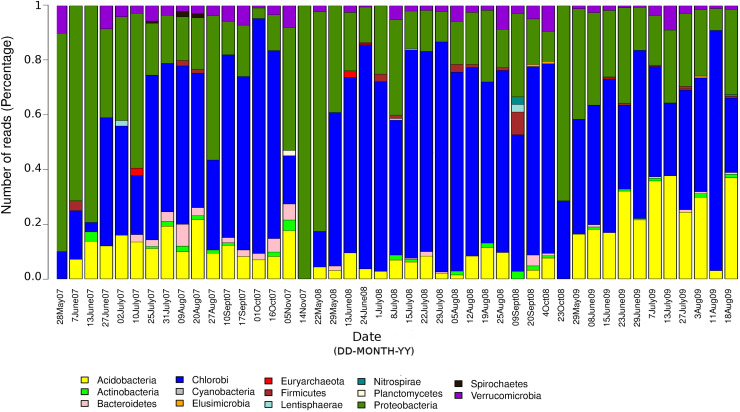
Relative contributions of different taxonomic groups to the potential diazotrophic community in Trout Bog Lake. The nitrogenase phylotype abundances were calculated using *nifH* reads identified from IMG annotations of assembled sequences and genomes using DNA data collected from hypolimnion.

The seasonal profile of nitrogenase gene abundances differed between the epilimnion and hypolimnion, suggesting contrasting environmental controls acting on N-fixers in the respective water masses (Figure 1A). For the hypolimnion specifically, diazotrophs were less abundant during May (early-middle spring) (Figure 1A). This suggests that hypolimnetic diazotrophs were poor competitors during this time of the year, which is generally characterized by spring mixing, a large influx of terrestrial matter from snowmelt, and gradual onset of thermal stratification. Associated with these overall dynamics in *nifH* relative abundance, we observed major changes in the taxonomic association of *nifH* genes as identified by IMG tools (Figure 2). After the spring mixing, *Proteobacteria* affiliates became abundant members of the *nifH* encoding sub-community. In contrast, *Chlorobi* growth seemed to be favored by stable stratification and truly anoxic conditions, prevailing from June to October in Trout Bog Lake.

Even if neither TN nor TDN alone were correlated to the seasonal shifts observed in the nitrogenase gene abundances, the prevalence of potential diazotrophs was favored by low TN:TP (<20). Interestingly, the growth of marine microorganisms in general reach an inflection point at N:P (=22) (average particulate ratio) (Martiny et al., 2014). Furthermore, active N-fixation seems to prevail in N-rich marine waters if P is also present and the ambient N:P is low (Bombar et al., 2016). So, how can aquatic diazotrophs stay competitive at high TN or TDN concentrations? Why would N-fixers maintain N-fixation ability while there is plenty of ammonia or nitrate available? These two questions remain unresolved, but there is growing evidence that N-fixation can stabilize and maintain an ideal intracellular redox state, a finding that decouples N-fixation from the function of exclusively satisfying the cellular N demand (Bombar et al., 2016).

### Freshwater and Marine Non-cyanobacterial Diazotrophs

The retrieved information about the seasonal *nifH* gene variance as an indicator of diazotroph abundances in Trout Bog Lake enabled us to perform a comparison between marine and freshwater microorganisms carrying the *nifH* gene beyond the well-studied diazotrophic *Cyanobacteria*.

Diverse sets of non-cyanobacterial nitrogenase gene phylotypes have indeed been described previously in both surface and deep marine waters (Farnelid et al., 2013; Bombar et al., 2016; Gradoville et al., 2017). In an extensive survey in the Pacific Ocean, the nitrogenase gene pool mainly consisted of cyanobacterial *nifH* phylotypes in the North Pacific, while *Alpha-* and *Betaproteobacteria* were very abundant across the depth gradient in the South and Northwest Pacific. Below the chemocline in the central Baltic Sea, nitrogenase gene and transcript libraries further showed that the N-fixer community consisted mainly of *Alpha*-, *Beta*-, and *Gammaproteobacteria* and diverse anaerobes including *Desulfovibrio* spp. and archaeal methanogens (Hsu and Buckley, 2009; Farnelid et al., 2013; Gradoville et al., 2017). This is different from what we observed in the hypolimnetic water mass of Trout Bog Lake, where *Chlorobi* dominated the potential diazotrophic communities. In agreement with our observations, *Chlorobi* and *Proteobacteria* were previously shown to be dominant in the sub-chemocline waters of meromictic Lake Cadagno during August, and in this setting, *Chlorobi* were also shown to be an active N-fixer based on isotope probing results (Halm et al., 2009). Further, *Chlorobi* was recently shown to be a major component of the NCD community also in permafrost thaw ponds, accompanied by members of phylum *Proteobacteria* (Fernandez et al., 2019).

In summary, we conclude that diazotrophs were less abundant during the spring period, possibly because of the large influx of terrestrial organics and nutrients from the snowmelt, which may have favored heterotrophs lacking *nif* operons. In addition, the prevalence of *nif* genes seemed to be coupled to photosynthetic bacteria, since *nifH* phylotype associated with *Chlorobi* were abundant in many samples (Figure 2). To the best of our knowledge, there are only a few studies (Halm et al., 2009; Thompson et al., 2017) demonstrating *in situ* diazotrophic potential in *Chlorobi*, and our results highlight that it may play a widespread significant role within diazotrophic humic lake communities.

### Nitrogenase Operon Structure and Accessory N-Fixation Proteins

**FIGURE 3:**
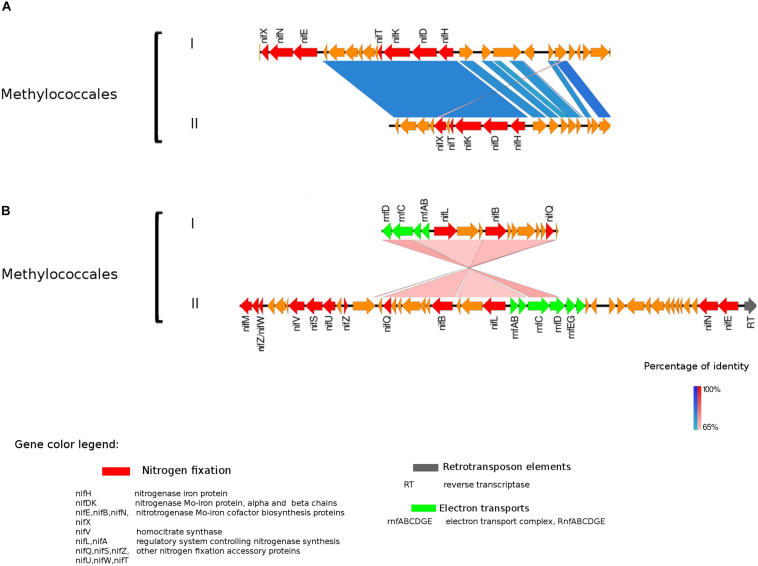
Alignment of nitrogenase gene clusters of two MAGs corresponding to potential N-fixers classified as *Methylococcales* (I) Composite genome 3552 [scaffolds: **(A)** TH3552DRAFT TBhypo metabat 3552 10001230.12, **(B)** TH3552DRAFT TBhypo metabat 3552 10006655.34] and Composite genome 2062v2 [scaffolds: **(A)** TH02062DRAFT TH02062 TBL comb47 HYPODRAFT 10005551.84, **(B)** TH02062DRAFT TH02062 TBL comb47 HYPODRAFT 10000828.15]. **(A)** The *nifHDK* gene cluster and **(B)** accessory N-fixation genes. Coding region sequences (CDS) are colored according to GenBank and KO annotation, with orange representing unknown or unclassified genes. Details of the general functions attributed to the CDS are listed in the color legend.

**FIGURE 4:**
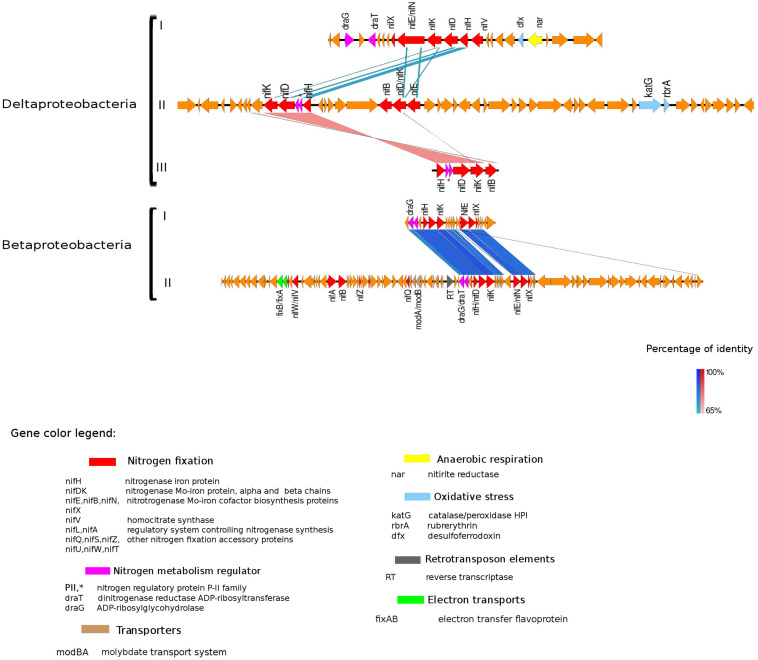
Alignment of nitrogenase gene clusters of five MAGs corresponding to potential N-fixers classified as *Deltaproteobacteria* (three scaffolds) and *Betaproteobacteria* (two scaffolds). *Deltaproteobacteria*: (I) Composite genome 4645 (scaffold: TH4645DRAFT TBhypo metabat 4645 10001890.329), (II) Composite genome 2922v2 (scaffold: TH02922DRAFT TH02922 TBL comb47 HYPODRAFT 10000417.24), (III) Composite genome 433 (scaffold: TH433DRAFT TBhypo metabat 433 10016042.145). *Betaproteobacteria*: (I) Composite genome 2160 (scaffold: TH2160DRAFT TBhypo metabat 2160 10003883.171), (II) Composite genome 2159 (scaffold: TH2159DRAFT TBhypo metabat 2159 10000110.67). Coding region sequences (CDS) are colored according to GenBank and KO annotation, with orange representing genes unknown or unclassified. Details of the general functions attributed to the CDS are listed in the color legend.

**FIGURE 5:**
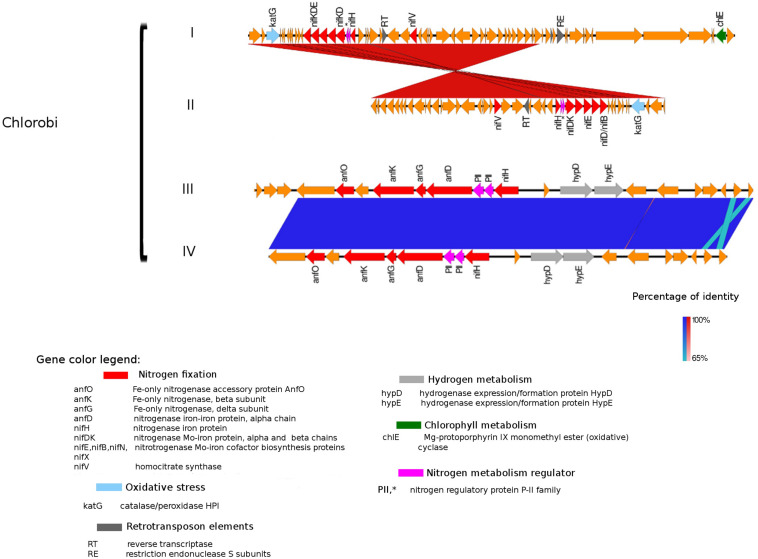
Alignment of nitrogenase gene clusters of four MAGs corresponding to potential N-fixers classified as *Chlorobi* (I) Composite genome 111 (scaffold: TH111DRAFT TBhypo metabat 111 10000140.1), (II) Composite genome 211 (scaffold: TE211DRAFT TBepi metabat 211 1000175.54), (III) Composite genome 2493 (scaffold: TE2493DRAFT TBepi metabat 2493 1001483.68), (IV) Composite genome 3520v2 (scaffold: TH03520DRAFT TH03520 TBL comb47 HYPODRAFT 10004746.48). In the upper panel (*Chlorobi* I and II) is represented the *nif* gene clusters and in the lower panel (*Chlorobi* III and IV) the *anf* gene clusters. Coding region sequences (CDS) are colored according to GenBank and KO annotation, with orange representing genes unknown or unclassified. Details of the general functions attributed to the CDS are listed in the color legend.

In prokaryotes, genes transcribed as an operon are usually regulated by a single promoter. Hence, genes within an operon typically belong to the same or related metabolic pathways (Sullivan et al., 2011). This genome architecture optimizes coordinated use of transcription factors and other regulatory elements in modulating the activity of the organism (Fernandez et al., 2014). Accordingly, we performed an analysis of the nitrogenase operon and surrounding genes (neighborhood analysis) with the purpose of obtaining more information about enzymes transcriptionally or metabolically related to the N-fixation process. Here, we included *nifH* gene clusters identified within the eight taxonomic groups of N-fixers mentioned above (only MAGs assembled from the seasonal metagenomic data from Trout Bog Lake were used in the analysis). The main genomic features surrounding the nitrogenase gene cluster were transcription factors involved in the regulation of nitrogen cycle processes, molybdenum transporters, enzymes protecting against oxidative stress, and energy metabolism-related proteins (Figures 3–5 and Supplementary Figure S2).

The best studied and most abundant nitrogenase in nature is the Mo-nitrogenase, encoded by *nifH, nifD*, and *nifK* genes, found in all N-fixers (Bothe et al., 2010). We observed the presence of *nifHDK* operon in 15 MAGs in Trout Bog Lake (see Figures 3–5 and Supplementary Figure S2). For the nitrogenase neighborhood analysis, only complete gene clusters were considered, which also included regulatory or accessory N-fixation proteins. For the iron-molybdenum cofactor (FeMoco) synthesis (Rubio and Ludden, 2008; Burén et al., 2019), additional genes *nifX*, *nifE*, *nifN*, *nifB*, and *nifV* may also be necessary (Chen et al., 2001). Those additional genes usually accompanied the *nifHDK* cluster (Figures 3–5 and Supplementary Figure S2), but were not universally present. In *Methylococcales* related genomes (Figure 3), extra copies of *nifE*, *nifN*, and n*ifB* were located downstream of accessory N-fixation genes: *nifQ*, *nifZ*, *nifU*, *nifS*, and *nifW*. These genes were, however, carried in a different gene cluster than *nifHDK* (Figure 3B). In addition to *Gammaproteobacteria*, accessory genes *nifQ*, *nifZ*, and *nifW* also appeared in betaproteobacterial diazotroph genomes, but in these cases, the genes were located near a cluster formed by *nifHDK*, *nifB*, *nifE*, and *nifN* (Figure 4). Other N-fixation accessory genes included *nifA* and *nifL* (Figures 3, 4), both associated as regulators (Barney et al., 2017) and in some *Proteobacteria*, their transcription is activated in response of oxygen concentration and fixed nitrogen availability (Martinez-Argudo et al., 2004).

An alternative enzyme for fixing nitrogen, the Fe-only nitrogenase, was present in genomes affiliated with *Chlorobi* (i.e., *Chlorobi* III and IV alignment in Figure 5). Fe-only nitrogenase as well as Mo-Fe nitrogenase were also found in *Rhodopseudomonas palustris*, *Rhodospirillum rubrum*, *Rhodobacter capsulatus*, and *Azotobacter vinelandii*, identified by Zheng et al. (2018). The Fe-only nitrogenase from these bacteria can use CO_2_, N_2_ gas and protons to produce CH_4_, NH_3_, and H_2_ gas in a single enzymatic step (Zheng et al., 2018). The broader relevance and distribution of methanogenesis/N-fixation properties attributed to the Fe-only nitrogenase was first suggested by Zheng et al. (2018). H_2_ is a common product of the Fe-only nitrogenase with methanogenic activity; interestingly, the Fe-only nitrogenase in the *Chlorobi* genome (comprised by *anfH/nifH*, and *anfOKGD* genes in Figure 5) is placed next to a hydrogenase domain. Based on this and earlier work (Zheng et al., 2018), we suggest that the Fe-only nitrogenase in *Chlorobi* (III and IV MAGs in Figure 5) may have potentially dual roles in both N-fixation and methanogenesis, also within the system studied here. The possible contribution of N-fixers carrying Fe-only nitrogenase in CH_4_ production in permafrost thaw ponds was also recently addressed by Fernandez et al. (2019) but no evidence of significant contribution of NCDs to elevated CH_4_ levels was found.

### Genetic Elements Related to Nitrogenase Expression and Activity

The presence of retrotransposable elements flanking the *nifHDK* genes in several freshwater populations (Figures 3–5) was a surprising finding, possibly indicating extensive horizontal gene transfer. For example, the nitrogenase cluster in *Chlorobi* (Figure 5) had many features of a retrotransposon. We found coding regions corresponding to reverse transcriptase (RT) and an endopeptidase as well as several direct and inverted repeats (Beauregard et al., 2008) and a predicted AAA-type ATPase (Arias-Palomo and Berger, 2015) (Figure 5). RT was also present in other Trout Bog MAGs (i.e., *Betaproteobacteria* and *Gammaproteobacteria*), but it was not possible to identify a complete set of additional and indispensable transposable element genes in these *Proteobacteria* (Figures 3, 4). Transposases near the *nif* cluster have been reported before (Xie et al., 2014). However, the dual role of transposons as “gold or trash” (Beauregard et al., 2008) makes the linkages between transposons and the inclusive genes noteworthy. Two non-exclusive hypotheses can explain the term “gold or trash” for a non-parasitic retrotransposon in bacteria. The first hypothesis states that genes carried on the retrotransposon may no longer be beneficial for the host and will eventually be lost during evolution. The second hypothesis emphasizes the role of the transposon in the regulation of gene expression, coupled with the ability to make extra copies of the desired genes if needed (Beauregard et al., 2008). We believe that both of these scenarios could be relevant for N-fixing ability on contemporary Earth.

Genes coding for post-translationally modifying proteins, such as the PII family and DraG/DraT, were also found adjacent to *nifH* or merely hundreds of base pairs away from the *nifHDK* cluster. The presence of *PII* regulator genes was characteristic for *Chlorobi* (Figure 5), and we also observed this feature in *Desulfobulbaceae* (*Deltaproteobacteria* II and III in Figure 4). In contrast, *draG* and *draT* genes were present in most representatives of *nifH* positive *Proteobacteria* (Figure 4), *Verrucomicrobia*, and *Acidobacteria* (see Supplementary Figure S2). The expression of these two important regulators of nitrogenase activity has been linked to ammonia availability (Bombar et al., 2016). According to the predicted function, PII family proteins, DraG (dinitrogenase reductase activating glycohydrolase) and DraT (dinitrogenase reductase ADP-ribosyltransferase) all can act as sensors for ammonia concentration and nitrogen limitation status (Chen et al., 2001; Oetjen and Reinhold-Hurek, 2009). Specifically, the target enzymes regulated by PII family proteins are directly involved in N metabolism pathways (Conroy et al., 2007) and can act as nitrogenase switch off in response to ammonia concentration (Kessler et al., 2001). While DraG and DraT proteins are modifiers of the nitrogenase enzyme (Nordlund and Ludden, 2006). DraT catalyzes the inactivation of nitrogenase by ribosylation and DraG can revert its functionality by removal of the ADP-ribosylation from the inactive Fe-Mo protein (Oetjen and Reinhold-Hurek, 2009) thus potentially representing a post-translational regulation system for N-fixation.

In the potential diazotroph genomes analyzed in Trout Bog Lake, carbon fixation genes were detected in most of them, and the regulatory *draT* accompanied *draG* gene was in the vicinity of the *nif* gene cluster in *Delta-* and *Betaproteobacteria* (Figure 4) and *Acidobacteria* and *Verrucomicrobia* (Supplementary Figure S2). Bombar et al. (2016) observed that either a disruptive mutation in the *DraT*, or the absence of this regulator in the presence of *DraG* coupled with an incomplete CO_2_ fixation pathway, could be associated with the reliance on N-fixation to maintain an ideal redox state in marine bacteria. In *Rhodobacter sphaeroides*, isolated from deep lakes, it was shown that electron-demanding nitrogenases could balance redox by producing dihydrogen as a byproduct of N-fixation (Kontur et al., 2011). Still, carbon fixation appeared to be the preferred reduction reaction accepting electrons. Thus, based on our genomic analysis, we hypothesize that if excess reducing power accumulates in the cell, hypolimnetic freshwater diazotrophs may expel the excess of reducing power via N-fixation.

In the hypolimnetic diazotroph communities analyzed here, we observed the presence of *nifL* in *Gammaproteobacteria* (Figure 3) and *nifA* in *Betaproteobacteria* (Figure 4). The *nifA* and *nifL* genes were found next to coding regions for electron-transfer and accessory N-fixation proteins in these Trout Bog MAGs. Both NifL and NifA are well known regulatory proteins affecting the nitrogenase gene expression. NifL acts as anti-activator of NifA, while NifA exerts a direct action on the nitrogenase promoter and increase *nifH* gene expression. In general, *nifL* and *nifA* gene expression responds to environmental and cellular signals such as energy, nitrogen, oxygen, and carbon availability (Barney et al., 2017). The action of NifA has also been linked to the transcriptional activation of genes encoding electron transport proteins (i.e., *fixAB* and *rnf*). NifA could be involved simultaneously in two process: (i) enhancing N-fixation by increasing nitrogenase expression and (ii) generating ATP by increasing electron transports expression and activity. Thus, the increased ATP yield could be used as fuel for nitrogenase enzyme activity (Sperotto et al., 2004; Barney et al., 2017).

Besides being broadly toxic to microorganisms, various reactive oxygen species (ROS) (i.e., hydrogen peroxide, superoxide) can also inhibit nitrogenase activity (Fay, 1992). Three different genes (*katG*, *rdrA*, and *dfx*), coding for enzymes involved in ROS and oxygen transformation, were found in the proximity of *nifHDK* gene clusters in several lineages analyzed here (Figures 4, 5).

In *Verrucomicrobia* and *Betaproteobacteria*, genes *modA* and *modB*, required for molybdate uptake, were also interspersed with N-fixation genes (Figure 4 and Supplementary Figure S2). In *Acidobacteria*, the scaffold containing the *nifHDK* cluster also had regions classified as amino acid transporters (PAAT), surrounded by genes involved in carbon metabolism (Supplementary Figure S2).

In summary, within the eight main taxonomic groups identified, *nif* operonic structure presented different characteristics. This suggests that diverse mechanisms may be involved in regulating nitrogenase transcription in Trout Bog diazotrophs. As N-fixation is energetically costly and diverts reducing power and ATP from other functions, cells have much to gain from precise and robust regulation of the expression and activity of nitrogenases. In agreement with this, the nitrogenase cluster (neighborhood) analyses suggested tight control of the *nif* operon. Nevertheless, with only genomic data, we cannot assure that the microorganisms carrying the genes for N-fixation actually use this metabolic option under the prevailing conditions.

### Reconstruction of the Central Pathways Related to Energy Metabolism Within Potential N-Fixers

Expanding our analysis to the whole genome is crucial to evaluate how the main diazotrophic groups deal with the high energy demand needed to perform N-fixation. Hypolimnetic waters of Trout Bog and other humic lakes are characterized by very low irradiance and suboxic to anoxic conditions (Watras et al., 2015). By inspecting the genomes of putative N-fixers, we encountered several options to generate or preserve metabolic energy (ATP) for N-fixation. Here, we limited the analysis to the main taxa highlighted in Supplementary Figure S1 to uncover metabolic strategies associated with N-fixation in autotrophic and heterotrophic potential diazotrophs. Taking into account *nifH* gene frequencies, *Chlorobi*, *Acidobacteria*, and *Proteobacteria* were the most abundant phyla during the sampling period. Therefore, we focused the metabolic analysis on the functional annotation of Trout Bog MAGs affiliated with these three groups, representing in total eight MAGs. The estimated completeness for the MAGs included in this analysis is reported in Table 2.

#### Chlorobi

Following the *nifH* operon and surrounding genes analysis, we started with the reconstruction of bacteriochlorophyll and carotenoid biosynthesis pathways. Both of these pigments are necessary for assembling the chlorosome, which constitutes the central structure for anoxygenic photosynthesis within *Chlorobi*, *Chloroflexi*, and *Acidobacteria* (Pšenčik et al., 2014). The genes involved in chlorosome formation and the central transformations in which their coding proteins participate are illustrated in Supplementary Figure S3. Only one essential gene, *bciC* (EC 3.1.1.100 enzyme in Supplementary Figure S3), related to bacteriochlorophyll c and d synthesis, was missing in the four MAGs affiliated to *Chlorobi*, in spite of the high percentage of genome completeness reported by JGI-IMG (from 89 to 94%, Table 2). Thus, the existence of an almost complete pathway to assemble the chlorosome supports photosynthetic potential in hypolimnetic *Chlorobi* by means of anoxygenic photosynthesis (Bryant et al., 2012) a process that would be feasible in the environmental conditions seen in the deeper waters of our stratified freshwater system (Xiong et al., 2000). Hydrogen could be an alternative energy source, implicated by the presence of hydrogenase genes. Such a process may also contribute to the removal of H_2_ and oxygen from the active site of the nitrogenase (Brewin, 1984). The presence of *dsrAB* genes suggests that *Chlorobi* can use sulfide as electron donor. DsrAB protein or dissimilatory sulfite reductase is a reversible system that functions in sulfite reduction and sulfur/sulfide oxidation. In the *Chlorobi* MAGs, we did not identify other enzymes to complete the dissimilatory sulfate reduction pathway, supporting our interpretation that *dsrAB* may play a role in sulfide oxidation (Müller et al., 2015). Trout Bog *Chlorobiales* MAGs also contain coding regions for key enzymes involved in the reductive tricarboxylic acid (rTCA) cycle (Linz et al., 2018) suggesting the use of CO_2_ as carbon source. Thus, based on the genomic analyses presented here, the possible mechanisms for electron flow in *Chlorobi* are photosynthesis, carbon fixation, and sulfide oxidation.

#### Acidobacteria

Despite a previous report of potential for chlorosome synthesis in this phylum (Pšenčik et al., 2014), we found no traces of phototrophic ability in the genome of *nifH* encoding hypolimnetic *Acidobacteria*. On the other hand, this genome hosted efficient carbon utilization and glycolate metabolism genes indicated by the presence of the *cstA* (carbon starvation), *coxL* and *cutL* (carbon fixation), and *glcD* (utilization of glycolate) in the vicinity of the nitrogenase gene cluster (Supplementary Figure S2). In a proteomic study of a soil that was rich in different *Acidobacteria* strains, coxL protein and different enzymes degrading small C1 compounds were found to be abundant. High prevalence of C1 metabolism in genomes from soil, including *Acidobacteria*, is believed to serve as support for larger enzyme complements involved in complex carbohydrate degradation (i.e., pectin and xylane) (Diamond et al., 2019). The most prominent carbon-related metabolic pathways we found in the *nifH* encoding *Acidobacteria* were the Arnon-Buchanan cycle, C4-dicarboxylic acid cycle, incomplete rTCA cycle, reductive pentose phosphate cycle, and phosphate acetyltransferase-acetate kinase pathway. In terrestrial environments, some strains of *Acidobacteria* appear to have genes for anaplerotic carbon dioxide fixation, a convenient function considering soils harbor pockets of high CO_2_ concentration (Eichorst et al., 2018). In summary, *nifH* encoding *Acidobacteria* in Trout Bog Lake seem to obtain energy and carbon by carbohydrate degradation; however, a principal energy source could not be identified.

#### Gammaproteobacteria (Methylococcales)

In *Proteobacteria* representatives, genes encoding bioenergetic enzymes, such as electron transport chain (ETC) component and the *Rnf* complex, were located in the neighborhood of nitrogenase related CDS (Figure 3). The *Rnf* complex has previously been found in *Methylococcales* (Chistoserdova, 2015). This complex, also detected in *Desulfobacterales* among the Trout Bog MAGs, is driving reverse electron flux from NADH, thus fueling energy demanding processes in the cell, such as N-fixation in *Clostridium ljungdahlii* (Tremblay et al., 2013). Thereby, it may provide electrons to the nitrogenase enzyme by contributing to ferredoxine reduction (Tremblay et al., 2013). Furthermore, there are reports that the Rnf complex is involved in the transcriptional and post-translational regulation and maturation of the nitrogenase enzyme in *A. vinelandii* (Curatti et al., 2005) and *Azoarcus* sp. *strain BH72* (Sarkar et al., 2012). We observed the presence of *rnf* complex genes placed among N-fixation related genes and regulators in *Methylococcales* (Figure 3), and in light of this, we suggest a possible role of *rnf* genes in the transcriptional and post-translational regulation of nitrogenase. Similar nitrogenase cluster organization and related genes arrangements were previously reported in *A. vinelandii* (Curatti et al., 2005) and *Azoarcus sp.* strain BH72 (Sarkar et al., 2012).

Genes indicating methylotrophy (*pmoA*, *mdh1*, *mxaF*) were identified in *nifH* encoding *Methylococcales* MAGs, indicating that C1 compounds could be a carbon and energy source. In addition, the use of C1 compounds as electron donor would be coupled with the Rnf complex to transfer reducing power to the nitrogenase reaction.

#### Deltaproteobacteria

Within the *Desulfobacterales* group, dissimilatory sulfate reduction seems to be the respiratory option considering the presence of *dsr* genes. This was combined with the *rnf* complex that can drive reverse electron flux (Tremblay et al., 2013) when coupled with energetically poor reaction such as sulfate reduction. This could provide electrons or reduced power in the form of NAD(P)H to fuel cytoplasmic [NiFe] and [FeFe] hydrogenases, formate dehydrogenases, and heterodisulfide reductase-related proteins (Pereira et al., 2011). The other representative of *Deltaproteobacteria*, *Geobacter*, did not contain any genes for dissimilatory sulfate reduction. Essential genes involved in the Wood-Ljungdahl (WL) pathway were found in both *Desulfobacterales* and *Geobacter*. In the Trout Bog MAG affiliated with *Geobacter* (Composite genome 4645), genes likely to be involved in extracellular electron transfer coupled with humic substances and Fe(III) reduction have been detected in a previous work (He et al., 2019). The carbon source (or an alternative energy source) detected in *Desulfobacterales* and *Geobacter* may be propionate or butanoate, as genes involved in short-chain fatty acid metabolism were found in these MAGs. Thus, according to our analysis, it seems that the main difference within *nifH* encoding *Deltaproteobacteria* is the electron sources: Fe(III) and humic substances in *Geobacter*, while *Desulfobacterales* seems to rely on sulfur compounds and Rnf complex to obtain electrons needed to feed the nitrogenase enzyme.

### Ecology of Microbial N-Fixers in Freshwater Hypolimnetic Waters

Analysis of freshwater *nifH* positive MAGs in this study enabled us to metabolically characterize previously undescribed potential diazotrophs from which we identified several possible metabolic strategies that may fuel N-fixation in the hypolimnion of Trout Bog Lake. The negligible abundance of *Cyanobacteria* (Linz et al., 2018) suggests that the characteristic environmental conditions of hypolimnetic waters, such as low irradiance, clearly prevented their growth and also likely N-fixation, which relies on energy from the canonical oxygenic photosynthesis via photosystems I and II (Heinz et al., 2016). While there is so far no data supporting that light may be an energy source for non-cyanobacterial but pelagic marine diazotrophs (Bombar et al., 2016), earlier work in meromictic Lake Cadagno suggests that in low irradiance and oxygen depleted hypolimnetic freshwaters, anoxygenic photosynthesis may provide the energy needed for N-fixation (Halm et al., 2009). Our genomic data support this hypothesis. For example, *Chlorobi*, the predominant hypolimnetic diazotrophic phylotype in Trout Bog Lake, have iron-sulfur-type photosystems enabling ferredoxin reduction and subsequent nitrogenase reduction to carry out N-fixation (Madigan, 2014). This alternative unique antenna system, the chlorosome, makes it possible for *Chlorobi* to obtain energy from a light-harvesting apparatus under low-irradiance and anoxic/suboxic conditions (Oostergetel et al., 2010). This structure constitutes an adaptation to fuel N-fixation at irradiances that are much lower than required for the cyanobacterial photosystems. *Chlorobi* was also found to be a major organism contributing to N-fixation in permafrost thaw ponds, further supporting this hypothesis (Fernandez et al., 2019). We also found carbon fixation genes (i.e., rTCA cycle and incomplete Calvin cycle) in *Chlorobi*, supporting the existence of a potential photosynthetic pathway that can be coupled with sulfide as an electron donor (Bryant et al., 2012). Our observations of genes likely encoding enzymes involved in photosynthesis, N-fixation, and carbon fixation in an anaerobic freshwater bacterium extend the potential for primary production deeper into the water column than previously assumed. Nevertheless, this needs to be confirmed with activity tests using stable nitrogen isotopes and transcriptomic analysis.

In Trout Bog Lake, the overlying epilimnetic waters are in contact with the atmosphere and profoundly influenced by aerobic photosynthesis, whereas the underlying hypolimnetic waters typically feature much less dissolved oxygen and become seasonally anoxic in summer. In this sense, hypolimnetic waters in Trout Bog Lake are representative and can be regarded as a model for the significant number of smaller and colored lakes in temperate and boreal regions. The depletion of oxygen is a determining step, as oxygen is a well-known disruptive factor for N-fixation; it can inhibit the nitrogenase enzyme activity, but it also represses *nifH* gene transcription (Fay, 1992). Thus, the inevitable exposure to oxygen has forced aerobic diazotrophs to develop strategies to couple processes such as the reduction of nitrogen with photosynthesis, as is the case of *Cyanobacteria* (Berman-Frank et al., 2003). Even if the hypolimnetic waters of Trout Bog Lake rapidly become anoxic after stratification sets in during spring (Linz et al., 2017), we observed the presence of three genes involved in the protection of bacterial cells from oxidative stress and ROS (Figures 4, 5). This may shield the hypolimnetic N-fixers from occasional exposure to oxygen-rich environments, which happens during seasonal overturn or episodic mixing events. There are plenty of other abiotic factors, such as molybdenum availability, that are known to regulate nitrogenase expression and activity. Although Mo is rarely limited, the most abundant nitrogenase is the Mo-containing protein, as this element is required for the assembling of the enzyme. In *Betaproteobacteria* and *Verrucomicrobia*, we accordingly observed the presence of Mo transport genes (*modAB*) in the neighborhood of the *nifHDK* cluster. For all other potential diazotrophs identified in our dataset, *modAB* were also present but located elsewhere on their reconstructed genomes. In a well-studied N-fixer, *Clostridium pasteurianum*, *modA* and *modB* genes were interspersed with N-fixation related genes. This is further support for co-regulation of molybdenum transport and N-fixation (Chen et al., 2001). Notably, *modAB* gene mutations affect nitrogenase activity negatively in *Klebsiella pneumoniae*, as they required higher levels of molybdate in the medium to fix nitrogen (Imperial et al., 1985).

The use of alternative terminal acceptors for the ETC is crucial for chemotrophic bacteria to mobilize energy from reduced organic compounds in oxygen-depleted waters efficiently. A complete pathway to carry out sulfate reduction was detected in *Desulfobacterales*. The use of sulfate as an electron acceptor for microorganisms is associated with reduced energy yield compared to use of oxygen, and other more favorable electron acceptors (Muyzer and Stams, 2008) which can partly be compensated by the availability of high-quality organic substrates. In freshwaters, it has been shown that sulfate reducing bacteria can also grow by fermentation using organic compounds as both reductant and oxidant (Muyzer and Stams, 2008). Freshwater sulfate reducing bacteria can also be syntrophically associated with methanogens and oxidize lactate and ethanol, while the hydrogen produced is efficiently removed by methanogenesis (Muyzer and Stams, 2008). In the *Desulfobacterales* MAGs analyzed here, a complete set of genes involved in the transformation of pyruvate to acetate was present. The WL pathway, which has been described before in organohalide-respiring *Dehalococcoides* (Zhuang et al., 2014), appeared to be present in these *Desulfobacterales* MAGs (complete in Composite genome 433 and incomplete in Composite genome 2922v2). The WL pathway can contribute to carbon fixation or energy conservation, depending on the microorganism’s physiological requirements (Zhuang et al., 2014). In the case of the WL pathway acting in reverse, generated reducing power from the oxidation of organic compounds may, at least in theory, fuel N-fixation.

## Conclusion

We uncovered an abundant and diverse community of non-cyanobacterial microorganisms in hypolimnetic lake waters that seems to have the potential to perform N-fixation. Potential diazotrophs were less abundant during the spring period, possibly because of the large influx of terrestrial organics and nutrients from the snowmelt, which may have favored heterotrophs lacking *nif* operons. In addition, the prevalence of *nif* genes seemed to be coupled to photosynthetic bacteria, since *nifH* phylotype associated with *Chlorobi* were abundant in many samples (Figure 2). To the best of our knowledge, there are only a few studies (Halm et al., 2009; Thompson et al., 2017) demonstrating *in situ* diazotrophic potential in *Chlorobi*, and our results highlight that it may play a widespread and significant role also within diazotrophic humic lake communities. Strategies for carbon fixation varied from WL pathway in *Deltaproteobacteria* to rTCA and reductive pentose phosphate cycle in *Acidobacteria*, while carbon fixation genes linked to photosynthesis were detected in *Chlorobi*. *NifH* positive MAGs classified as *Chlorobi* are an example that primary production zone may expand to deep water layers in Trout Bog Lake. In summary, carbon fixation and utilization pathways could be linked to N-fixation in the hypolimnion of Trout Bog Lake, and we propose that this finding extends to other humic and stained freshwater systems. In contrast, we observed marked differences in the energy metabolism capabilities regarding terminal electron acceptors and electron transport proteins within the diazotrophic community. We also infer a potential relationship between nitrogenase expression and the *rnf* complex (electron carrier proteins) in *Methylococcales*; supported by the structure of the operons featuring *rnf* and N-fixation related genes. Ongoing metatranscriptomic studies in the same bog system may help clarify these linkages and reveal the realized metabolism of the potential diazotrophs identified in this research. Nevertheless, post-translational regulators as identified in our genomic study point to limitations of transcriptomics when studying highly regulated metabolic processes and call for future coordinated analyses of metaproteomes and N-fixation rates.

## Data Availability Statement

The datasets generated for this study can be found in the Joint Genome Institute Genome Portal under the JGI Identification number 416375.

## Author Contributions

SB conceived this study and KM supervised the environmental data collection, seasonal experiments, and DNA sequencing protocols. LF analyzed the data, created the main figures, and also drafted a first version of the manuscript. LF, AE, SP, and SB designed the methodologies to analyze N-fixers and analyzed the results. AL contributed with the laboratory work and sampling. All authors have been involved in writing the manuscript as well as analyzing and discussing the results. All authors have read and approved the final version of the manuscript.

## Conflict of Interest

The authors declare that the research was conducted in the absence of any commercial or financial relationships that could be construed as a potential conflict of interest.

## Supplementary Material

FIGURE S1 Phylogenetic tree of the potential diazotrophs inhabiting the hypolimnetic waters of Trout Bog Lake. MAGs from Trout Bog Lake containing a *nifH* gene sequence are included in the tree. Different colors highlight the most abundant groups of possible diazotrophs. For the genome name, we used a shortened version of the IMG full name (i.e., IMG full name: Composite genome from Trout Bog Hypolimnion pan-assembly Tbhypo.metabat.254; short name: Composite genome 254). The numbers above the branches represent the branch length and the numbers inside the blue circles represent the length in the ramification. FastTree tool was used to build the tree. FastTree computes large minimum evolution tree with profiles instead of a distance matrix and was included in the PhyloPhlAn pipeline.

FIGURE S2 Alignment of nitrogenase gene clusters of four MAGs corresponding to potential nitrogen fixers classified as Acidobacteria (1 scaffold), Sulfurimonas (1 scaffold), Verrucomicrobia (2 scaffolds) (see Material and Methods for more information about the scaffolds used). Coding region sequences (CDS) are colored according to GenBank and KO annotation, with orange representing genes unknown or unclassified. Details of the general functions attributed to the CDS are listed in the color legend.

FIGURE S3 Chlorosome formation and anoxygenic photosynthesis genes. The figure presents the central genes and transformations in which their coding proteins are involved. The colored squares represent the group of microorganisms that contains a homologous gene for the illustrated function. The same color legend as in Supplementary Figure S1 is used to highlight the different phylogenetic categories.
