# Supplementary material for: Diazotroph Genomes and Their Seasonal Dynamics in a Stratified Humic Bog Lake
Source: Front Microbiol. 2020 Jul 1;11:1500. doi: 10.3389/fmicb.2020.01500 (PMC7341956; doi:10.3389/fmicb.2020.01500)
Supplement: Supplementary file 4 [file Table_1.DOCX]

Supplementary figures:

Supplementary Figure 1:Phylogenetic tree of the potential diazotrophs inhabiting the hypolimnetic waters of Trout Bog Lake. MAGs from Trout Bog Lake containing a *nifH* gene sequence are included in the tree. Different colors highlight the most abundant groups of possible diazotrophs. For the genome name, we used a shortened version of the IMG full name (i.e., IMG full name: Composite genome from Trout Bog Hypolimnion pan-assembly Tbhypo.metabat.254; short name: Composite genome 254). The numbers above the branches represent the branch length and the numbers inside the blue circles represent the length in the ramification. FastTree tool was used to build the tree. FastTree computes large minimum evolution tree with profiles instead of a distance matrix and was included in the PhyloPhlAn pipeline.

Supplementary Figure 2: Alignment of nitrogenase gene clusters of four MAGs corresponding to potential nitrogen fixers classified as Acidobacteria (1 scaffold), Sulfurimonas (1 scaffold), Verrucomicrobia (2 scaffolds) (see Material and Methods for more information about the scaffolds used). Coding region sequences (CDS) are colored according to GenBank and KO annotation, with orange representing genes unknown or unclassified. Details of the general functions attributed to the CDS are listed in the color legend.

Supplementary Figure 3: Chlorosome formation and anoxygenic photosynthesis genes. The figure presents the central genes and transformations in which their coding proteins are involved. The colored squares represent the group of microorganisms that contains a homologous gene for the illustrated function. The same color legend as in Supplementary Figure. 1 is used to highlight the different phylogenetic categories.
